# Editorial: Advances in the Systemic Therapy and Combined Modality Approaches for Head and Neck Cancer

**DOI:** 10.3389/fonc.2019.01190

**Published:** 2019-11-13

**Authors:** Athanassios Argiris, Lisa Licitra

**Affiliations:** ^1^Thomas Jefferson University, Philadelphia, PA, United States; ^2^Istituto Nazionale Tumori, Milan, Italy

**Keywords:** systemic therapy, chemotherapy, cetuximab, head and neck cancer, squamous cell carcinoma of the head and neck, human papillomavirus

In the past few years there have been several developments in the field of head and neck oncology, including major advances in systemic therapy and combined modality treatment. Systemic therapy indeed has been playing an increasing important role in the management of squamous cell carcinoma of the head and neck (SCCHN) in both the locally advanced and the recurrent/metastatic disease settings.

This Special Issue of Frontiers in Oncology focuses on “Advances in the Systemic Therapy and Combined Modality Approaches for Head and Neck Cancer” and was compiled with the main objective of providing a timely overview of emerging concepts in the systemic therapy of head and neck cancer and the integration of systemic agents into multimodality management. We are very thankful to have received the contributions of many prominent experts in head and neck oncology. The Special Issue encompasses reviews on combined modality approaches in locally advanced SCCHN, including cisplatin eligibility issues, postoperative treatment, and laryngeal preservation approaches, an original report of an induction trial with cisplatin, docetaxel, cetuximab followed by radiotherapy and cetuximab, as well as reviews of the current and upcoming role of targeted therapies and immunotherapy in SCCHN. The role of cetuximab in recurrent or metastatic SCCHN is reviewed as well. Combinations of a taxane and cetuximab are active in the first-line setting and have been evaluated in randomized trials. We also cover therapeutic developments in nasopharyngeal cancer and include reviews on prognostic factors in recurrent or metastatic SCCHN and nasopharyngeal cancer that are potentially relevant for patient assessment and treatment decisions. Another interesting review focuses on emerging treatment strategies in human papillomavirus-positive oropharyngeal cancer, an increasing subset of SCCHN with different biology and better treatment outcomes.

We hope that this Special Issue of Frontiers in Oncology succeeded in stimulating the interest of our readers in systemic therapy options for the management of head and neck cancer. As the field is evolving our efforts will continue in order to provide updates with emerging data.

## Author Contributions

All authors listed have made a substantial, direct and intellectual contribution to the work, and approved it for publication.

### Conflict of Interest

The authors declare that the research was conducted in the absence of any commercial or financial relationships that could be construed as a potential conflict of interest.

